# Reverse Genomics: Design of Universal Epitope Sets to Isolate All *Saccharibacteria* Members from the Human Oral Cavity

**DOI:** 10.3390/microorganisms10030602

**Published:** 2022-03-11

**Authors:** Ahmad Ibrahim, Mohamad Maatouk, Didier Raoult, Fadi Bittar

**Affiliations:** 1 IHU Méditerranée Infection, 13005 Marseille, France; ahmad.ibrahim@etu.univ-amu.fr (A.I.); mohamad.maatouk@etu.univ-amu.fr (M.M.); didier.raoult@univ-amu.fr (D.R.); 2 Aix-Marseille Université, IRD, APHM, MEPHI, 13005 Marseille, France

**Keywords:** reverse genomics, candidate phyla radiation, universal epitopes, co-culture, *Saccharibacteria*

## Abstract

Microorganisms not yet cultured represent a large proportion of the microbes described to date. Progress in sequencing and metagenomic tools continues to increase microbial diversity without providing information on their physiological and pathophysiological characteristics, such as the recent discovery of enigmatic microbes belonging to Candidate Phyla Radiation (CPR). Reverse genomics is a recent technique allowing co-cultivation of a few CPR members, affiliated to the *Saccharibacteria* phylum, based on the analysis of their already-available genomes. Here, our aim is to designate a common system capable of cultivating any given taxon of this phylum from human samples. We managed to design, in silico, 11 common epitopes for all *Saccharibacteria* species recovered from the human oral cavity and which can serve as antigens via bioinformatics analyses. These sequences allow the synthesis of target antibodies, sorting *Saccharibacteria* spp. by flow cytometry and co-culturing them afterwards with adapted hosts. This epitope set can facilitate the cultivation of CPR in general, which in recent years has been considered a challenge for microbiologists, and subsequently contributes to better studying this new branch on the tree of life.

## 1. Introduction

The development of high-throughput sequencing methods and continual metagenomic explorations have paved the way to many findings and discoveries in the 21st century [1]. These intensive efforts have allowed microbiologists to improve the characterisation of the human microbiome, investigate microbial diversity in different ecosystems, and discover new microbial species and divisions [2,3]. Recently, progress with these tools has enabled scientific communities to define for the first time, in 2015, a neglected microbial division, close to the bacterial domain, but quite unique, named Candidate Phyla Radiations (CPRs) [3,4]. This nomenclature has been attributed to these microbes, given that all their members are uncultivated axenically at this time [3,5].

CPRs are considered to be mini microbes due to their small size (ranging from 100 to 300 nm) [6]. In addition, their genome is also reduced compared to standard bacteria (mainly less than 1 Mgb) [7]. These microorganisms present a particular lifestyle, represented by an obligatory physical attachment between them and a host cell (most often bacterial hosts), which could be either an exo-symbiotic or exo-parasitic relationship [3,6]. This obligatory relationship is supported by the presence of a type IV pili-like system at the level of the CPR outer cell membrane, which allows their attachment to the bacterial cell wall [6]. In addition, recent studies have shown their rich repertoire of Quorum sensing proteins and signals, which facilitates microbial cell to cell dialect (CPR–host communication) [8,9].

Moreover, thanks to different metagenomic studies, CPRs seem to have a possible clinical involvement. For example, *Saccharibacteria* (the most studied CPR phylum) is associated with inflammations of the oral mucosa such as periodontitis, gingivitis, and halitosis [10,11]. Moreover, it is suggested that the abundance of CPRs in the human colon (more specifically *Saccharibacteria* and *Parcubacteria* phyla) causes an alteration of the mucosal layers, which leads to gastrointestinal dysfunction, as well as different chronic inflammatory disorders such as bowel and Crohn’s diseases [12]. The prevalence of *Saccharibacteria* has also been associated with some infectious diseases as well, such as infections by *Helicobacter pylori* and *Schistosoma japonicum* [13,14].

To date, most CPR characteristics are only predicted from genomic analyses. Therefore, CPR member culturing is a critical and indispensable step to better understand their physiology and physiopathology [15]. This process remains a major challenge for microbiologists, who are developing different protocols and techniques to cultivate fastidious and not-yet-cultured bacteria and, more precisely, CPR cells [7,15,16,17,18].

Recently, a new approach called “Reverse genomics” has been developed by Cross et al. in 2019 for culturing not-yet-cultured bacteria [19]. This technique is based on the use of specific antibodies targeting transmembrane proteins of the cell of interest, in order to sort it by flow cytometry and subsequently culture it in a suitable medium. The choice of epitopes targeted by the antibodies is based on the total analysis of a given genome (the amino acid sequences of the strain of interest). This methodology allows the cultivation of any microbe with an available genome recovered from metagenomes of any environment and facilitates their subsequent phenotypic characterisation. [19]. In the study of Cross et al., a common epitope set for only two *Saccharibacteria* genomes was selected in order to synthesise target antibodies for them [19]. Then, flow cytometry sorting was performed for the relevant strains for subsequent cultivation of three *Saccharibacteria* strains and one human oral SR1 specimen [19].

Here, we are interested in developing an exhaustive capture that allows us to target the maximal possible number of *Saccharibacteria* species. The aim is to find a universal epitope set, specific but common to all *Saccharibacteria* species isolated from the human oral cavity.

## 2. Materials and Methods

For this purpose, we selected all *Saccharibacteria* complete genomes available on NCBI (National Centre for Biotechnology and Information) (https://www.ncbi.nlm.nih.gov, accessed on 9 March 2022) up to 4 October 2021. We focused our analysis only on good quality genomes sequenced from the human oral cavity (*n* = 20). Then, these genomes were annotated by Rapid Annotation using the Subsystem Technology tool kit (RASTtk) as implemented in the PATRIC v3.6.8 annotation web service [20].

Later, each protein sequence was split into fragments containing 20 amino acids, with an overlap of 10 amino acids, using the splitter online tool (Galaxy version) [21]. Then, a comparison of all generated fragments between the analysed genomes was performed using the Proteinortho program [22] and Diamond tool [23]. The used thresholds were a minimum identity of 100%, a minimum coverage of 50%, and a maximum e-value of 0.001. We performed this step in an attempt to obtain identical protein sequences (without gaps) of a size ranging between 10 and 20 amino acids, shared by all genomes. Afterwards, all proteins that contained these conserved amino acid regions were selected for the next step. These proteins were screened for the presence of transmembrane helical domains using the TMHMM v.2.0 online tool [24]. Potentially transmembrane proteins were visualised and confirmed by the Protter tool [25] and OPM database [26]. All proteins annotated as hypothetical proteins without a recognised domain (according to motif search) and those shorter than 100 amino acids were eliminated, exactly as previously described [19]. Next, in order to select peptides that could serve as antigenic determinants (with a size ranging between 10 and 20 amino acids), each remaining protein was analysed for its antigenicity, antibody accessibility (i.e., potential linear regions) and peptide hydrophilicity using different online tools: SVMTrip [27], BepiPred [28], and MINNOU [29].

After that, BLASTp analyses of the candidate epitopes were performed against HOMD (Human Oral Microbiome Database: http://www.homd.org, accessed on 7 March 2022) [30] in order to select specific antigenic determinants that only match with *Saccharibacteria* members. Finally, a three-dimensional structure (3D) of each selected protein was predicted using the Phyre2 online tool [31].

## 3. Results and Discussion

**Figure 1:**
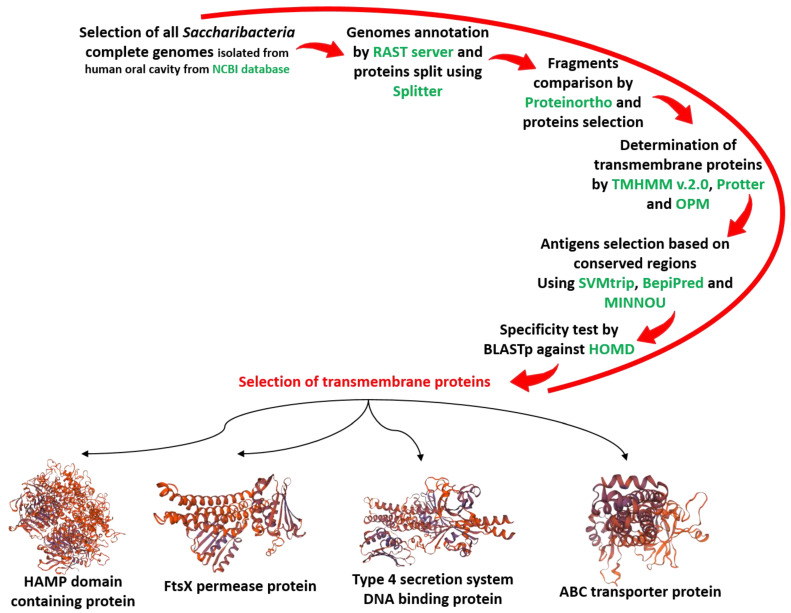
Study design showing the candidature proteins found. All bioinformatics pipelines used in this study are marked in green.

Following genome annotation, our *in silico* protein fragmentation yielded an average of 32,000 fragments/genome for further analyses. By comparing them to each other using Proteinortho [22], we noticed the presence of 389 sequences of 10–20 amino acids that are common among all tested genomes, distributed over 114 different proteins. After predicting the locations of these last proteins in the cell, only 16 of the 114 showed a transmembrane location. Finally, the analyses of these proteins showed that only 4 possess antigenic determinants, with linear and antibody-accessible sequences that are present in the outer part of these transmembrane proteins (Figure 1).

The first protein-encoding gene belongs to a type IV secretion system DNA-binding domain. In this protein, we detected seven peptides or epitopes (size ranging between 10 and 16 amino acids) serving as specific antigens for *Saccharibacteria* members. This protein is a part of the pili type IV secretion complex, which is considered to be an essential component for the life style of all *Saccharibacteria* members, supporting their attachment to the host bacteria [6]. The second protein codes for an FtsX permease protein; we could detect two epitopes with a size of 10 amino acids. This protein is part of the ABC transporter FtsEX, which is involved in bacterial cell division [32], according to UniProt. Moreover, in the third protein (HAMP domain containing protein), we detected one epitope with a size of 12 amino acids. It is known to be a protein present in the membrane of some prokaryotes and is involved with various signal transduction pathways [33]. Likewise, only one epitope with a size of 14 amino acids was detected in the last protein, which contains a PAS domain. This domain is involved in a protein–protein interaction [34,35]. All amino acid sequences (epitopes) are listed in Table 1.

Moreover, to determine the specificity of these sequences, we screened each predicted peptide against HOMD by BLASTp, and no similarity was detected with any other microbial species present in the human oral microbiota.

High-throughput sequencing has improved our knowledge of microbial diversity [2,19]. The absence of a pure representative member still prevents us from understanding the physico-chemical characteristics of a given organism and its interaction with other microbes [19]. The reverse genomics technique has been applied recently, showing the advantages of cultivating one of the fastidious microbes belonging to the *Saccharibacteria* phylum of CPR division with flow cytometry sorting [19].

In our study, we also succeeded in obtaining in silico specific epitopes for *Saccharibacteria* spp., and they are conserved in all described species from the human oral cavity. These peptides are distributed into four different transmembrane proteins. The use of antibodies targeting these epitopes can help in culturing new members of this phylum and enrich our knowledge about these abnormal microbes.

Furthermore, as of today, this methodology is considered the more suitable one to co-cultivate these microbes with the bacterial host they naturally associate with. Most studies on the CPR members co-culture is based on the filtration of the detached *Saccharibacteria* members from their natural host to co-cultivate them with hosts of choice for testing, such as *Schaalia odontolyticus* and *Arachnia propionica* [7,15,16,17,18]. However, thanks to reverse genomics, Cross et al. have described for the first time the interaction of a *Saccharibacteria* member with *Cellulosimicrobium cellulans* [19]. This suggests that the use of our epitope sets to synthesise antibodies increases the possible number of co-cultured *Saccharibacteria* cells, since our genomic analysis is not limited to one or two specific genomes. Moreover, this technique improves—in a pure, specific, and rapid way—the description of further clinical species of CPR, and subsequently shows their diversity and the types of bacteria that interact with them [19].

In addition, this strategy can also be applied to archaea, or other phyla/taxa, such as the Parcubacteria phylum and DPANN group (*Diapherotrites*, *Parvarchaeota*, *Aenigmarchaeota*, *Nanoarchaeota*, and *Nanohaloarchaeota*) of archaea [2]. The use of this methodology can be employed to fill in many gaps regarding not-yet-cultivated genome-available members in the tree of life [19].

Metagenomics studies have shown that the *Saccharibacteria* superphylum has a significant impact on human health [18,36]. In addition, different studies have shown that environmental *Saccharibacteria* spp. are genetically different from clinical/human ones [37]. Therefore, for clinical interest, we were only interested in available genomes of human origin. At the same time, our analyses were limited to complete genomes of good quality, according to PATRIC (https://www.patricbrc.org, accessed on 7 March 2022), to have the most exhaustive and specific genetic information from each genome. However, our study was unfortunately limited to prediction and specific bioinformatics designing, since flow cytometry is not available in our research laboratory. By in silico analysis, our epitopes are specific for *Saccharibacteria* (no similarity with other bacterial proteins was detected by BLASTp), and sensitive to all described species/genomes to date (100%). This does not negate the importance and the need to eventually use it to synthesise antibodies and test its specificity and sensitivity in vitro.

## 4. Conclusions

In conclusion, the application of this strategy can facilitate the cultivation/co-cultivation of the most fastidious microorganisms and contribute to the phenotypic characterization of many members of the microbial dark matter.

## Figures and Tables

**Table 1:** All selected epitope sequences.

	Protein Annotation	10 a.a	12 a.a	14 a.a	16 a.a
1	Type 4 secretion system DNA-binding domain-containing protein	LLELFALSDI	SGLLELFALSDI	APVLNKVGAFTANP	GKSGLLELFALSDIFH
			VNLSKGLIGEDN		AGKSGLLELFALSDIF
					ILGSFLVTKIQLAAMS
2	FtsX-like permease protein	PSKDEVEVEI			
		TFFTKGTKQL			
3	HAMP domain-containing protein		LAALRIMLENMQ		
4	PAS domain-containing protein			RLEHIFRNCALLLM	

## Data Availability

Not applicable.
